# The weight of genotype on the clinical presentation of *COQ7* -related hereditary motor axonal neuropathy: a case series and literature review

**DOI:** 10.1055/s-0046-1818609

**Published:** 2026-06-25

**Authors:** Laura Alonso Matheus Montouro, Amanda Selvátici Santos Dias, Maria da Penha Ananias Morita, Érica Nogueira Coelho, João Aris Kouyoumdjian, Carla Renata Graca, Fábio de Nazaré Oliveira, Pedro Henrique Marte Arruda Sampaio, Charles Marques Lourenço, Eduardo de Paula Estephan

**Affiliations:** 1 Faculdade de Medicina de São José do Rio Preto, Departamento de Ciências Neurológicas, São José do Rio Preto SP, Brazil.; 2 Universidade de São Paulo, Faculdade de Medicina, Departamento de Neurologia, São Paulo SP, Brazil.

**Keywords:** Charcot-Marie-Tooth Disease, Spastic Paraplegia, Muscular Atrophy, Spinal, Mitochondrial Diseases

## Abstract

**Background:**

Pathogenic variants of the *COQ7* gene result in a spectrum of neurological diseases, mainly distal hereditary motor neuropathy (dHMN). We herein report cases of dHMN related to biallelic p.Met1? (c.3G > T [NM_016138]). We compare phenotypes among different *COQ7* variants reported in the literature.

**Objective:**

To describe and analyze our case series, review *COQ7* -related diseases, and compare our case series with the literature reports.

**Methods:**

We described 5 dHMN-p.Met1? patients and searched *dHMN* AND *Brazil* and COQ7 in the PubMed/MEDLINE, SciELO and Scopus databases. The categorical variables were expressed as absolute frequencies, and they were compared using the Fisher's exact test and odds ratios with 95%CIs; moreover, exploratory multivariate logistic models with penalization were applied to adjust the associations for genotype/geographic origin.

**Results:**

We analyzed four patients with dHMN plus (two with pyramidal syndrome [PS], one with cerebellar ataxia [CA] and PS, and one with cognitive impairment [CI]) and one with pure dHMN. Our search identified 47 cases of *COQ7* -related disorders, and The p.Met1? variant was more frequent in dHMN ( *p* < 0.001). In exploratory multivariate models adjusting for genotype/geographic origin, the associations observed in the univariate analyses were partially sustained. The p.Met1? variant remained related to earlier age at onset, CI, and proximal lower limb weakness, whereas Brazilian origin continued to show association with cerebellar manifestations. The 95%CIs were wide due to the small sample, and the results should be interpreted as exploratory.

**Conclusion:**

In conclusion, our findings suggest that the p.Met1? variant is associated with selected phenotype, even after adjustment for genotype/geographic origin. Brazilian origin remained independently related to cerebellar involvement, indicating potential modifying factors beyond genotype.

## INTRODUCTION

Mitochondrial diseases can occur due to genetic changes in nuclear or mitochondrial DNA, leading to defects in oxidative phosphorylation. 1 2 Coenzyme Q10 (CoQ10) is essential as an electron transporter and antioxidant. 3 Consequently, CoQ10 is an important protein in cellular metabolism. 3 Primary CoQ10 deficiency is a rare disease characterized by genetic and phenotypic heterogeneity. 4 The condition is often treatable, usually responding to exogenous CoQ10 replacement, 4 and new therapeutic targets are also under study. 5 6 7 8 Several genes have already been correlated with primary CoQ10 deficiency in humans: *PDSS1* , *PDSS2* , *COQ8A* , *COQ8B* , *COQ7* , *COQ9* , *COQ6* , *COQ4* , *COQ6* , *COQ2* , and *COQ5* . 9

The mitochondrial enzyme 5-demethoxyubiquinone hydroxylase, encoded by the *COQ7* gene, is one of the enzymes involved in CoQ10 biosynthesis. 10 11 Mutations in *COQ7* result in primary CoQ10 deficiency-8 (CoQ10D8). 3 The first case of neurological disease related to *COQ7* was published in 2015. 6 It was a multisystemic form (MF) of the CoQ10 deficiency, with the mitochondrial encephalo-myo-nephro-cardiopathy phenotype. Subsequently, the *COQ7* phenotype was expanded to include spastic paraplegia (SPG), 5 10 12 13 14 demyelinating or axonal sensorimotor polyneuropathy, 10 12 14 15 and motor neuron disease. 16 In recent years, some case series 6 12 13 14 15 17 18 19 20 21 22 23 24 25 26 27 have been published associating *COQ7* variants with distal hereditary motor neuropathy (dHMN). Rebelo et al. 15 reported cases in nine families with *COQ7* -related dHMN, and five Brazilian families harbored the p.Met1? variant, which shows signs of upper motor neuron dysfunction, cognitive impairment, and cerebellar ataxia (CA). Evidence for a founder effect of the p.Met1? variant in the region was also provided by Rebelo et al. 15 We herein report the cases of 5 Brazilian patients from 3 unrelated families with the same p.Met1? (c.3G > T [NM_016138]) variant presenting different neurological findings associated with dHMN, reinforcing the link between this phenotype and the specific p.Met1? *COQ7* variant. We also provide a narrative review of *COQ7* disorders, comparing the phenotypes among different variants.

## METHODS

In order to conduct the present observational and descriptive study, we retrospectively collect a series of cases from the Neuromuscular Outpatient Clinic at the tertiary Teaching Hospital of Faculdade de Medicina de São José do Rio Preto (FAMERP), in the state of São Paulo, Brazil. All cases were submitted to a genetic panel for neuromuscular disease involving 110 genes and using next-generation sequencing with the Illumina technology (Illumina, Inc.). Variant alignment and identification were performed using standard bioinformatics protocols, referencing the Genome Reference Consortium Human Build 38 (GRCh38). All procedures in the current study were performed under the ethical standards of the FAMERP Ethics in Research Committee and in accordance with the 1964 Helsinki Declaration and its later amendments or comparable ethical standards.

We compared the clinical features with the reported *COQ7* -patients and Brazilian dHMN cohorts in the literature. In July 2025, the PubMed/MEDLINE, SciELO, and Scopus databases were searched using the terms COQ7 and *dHMN* AND *Brazil* . The results were screened according to the type of study, and we only selected those involving humans. The categorical variables were expressed as absolute frequencies and compared using the Fisher's Exact test and odds ratios (OR), with 95%CIs. All tests were two-tailed. Statistical significance was set at p < 0.05 for all two-tailed tests. Descriptive tables and statistical computations were conducted using Google Sheets (Alphabet Inc.) and VassarStats software.

## RESULTS

### Case series

**Figure 1:**
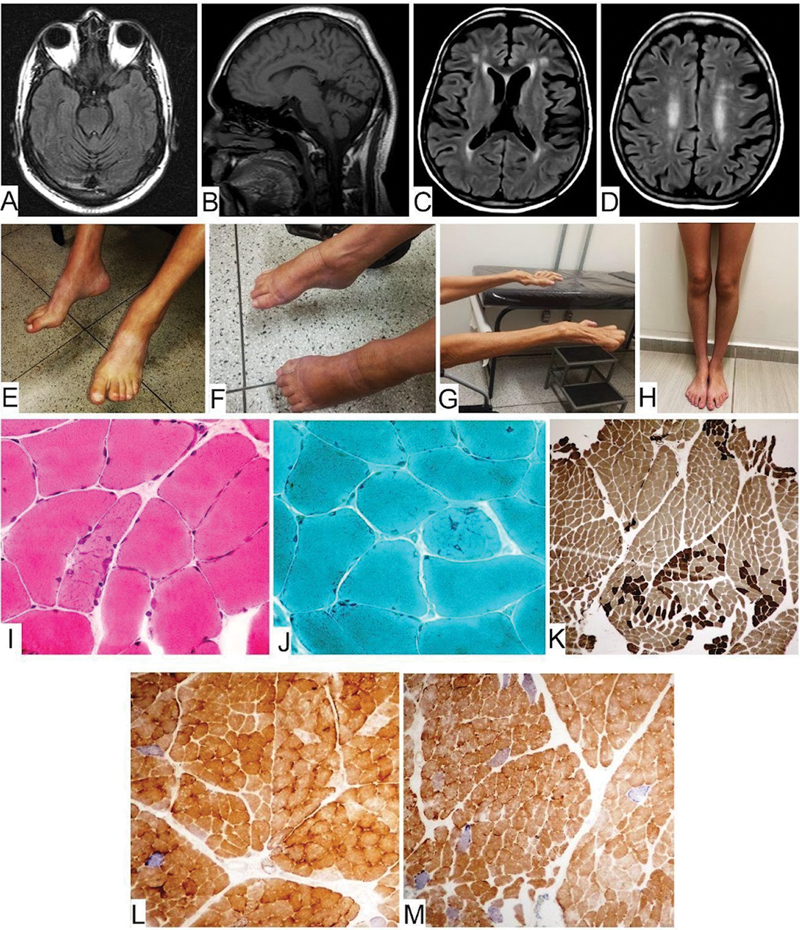
Brain magnetic resonance imaging (MRI) scan showing cerebellar atrophy, affecting predominantly the lobes: fluid-attenuated inversion recovery (FLAIR) sequence ( **A** : axial; **B** : sagittal) in patient 1. Brain MRI scan with microangiopathy disease (Fazekas 3 on periventricular and deep white matter) in patient 4 ( **C** , **D** ). Pes cavus: patient 1 ( **E** ). Muscular atrophy of the foot and hand: patient 4 ( **F** , **G** ). Patient 5: lower limb atrophy affecting the tibialis anterior muscle and the medial portion of the thigh ( **H** ). Muscle biopsy of Patient 3 showed variation in muscle fiber diameter, with scattered small fibers and fibers presenting internal nuclei; multiple granular fibers (hematoxylin and eosin) ( **I** ) and ragged-red fibers were also noted.” (Gomori's trichrome stain) ( **J** ). Patient 4's muscle biopsy: a predominance of type-1 fibers was observed in the reactions by adenosinetriphosphatases (ATPases), with some rare areas of type-2 fiber grouping without atrophy or hypertrophy (ATPase pH: 9.4) ( **K** ) Several cytochrome c oxidase (COX)-negative fibers were observed, demonstrating positivity on combined COX/succinate dehydrogenase (SDH) histochemistry.) in both cases ( **L** , **M** ).

Patient 1. A 40-year-old man, born to consanguineous parents, reported difficulty walking since childhood and sporadic falls during sports activities. Over time, he developed imbalance, bilateral incoordination in the upper limbs, slow speech, and the need for aid to walk and to perform basic activities of daily living, with more frequent falls. The physical findings included fixed contracture of the ankle tendons, high-arched feet ( Figure 1E ), slurred speech, a wide-based gait, and preserved muscle strength, except for weakness in ankle dorsiflexion. The knee reflexes were increased, and those of the ankle were abolished. Appendicular ataxia, trunk-limb incoordination, and hypometric saccades were also noted. The mental status was preserved. A brain Magnetic Resonance Imaging (MRI) scan ( Figure 1A,B ) revealed mild cerebellar atrophy with a predominant involvement of the vermis and the upper half of the cerebellar hemispheres. Neurophysiological studies were consistent with motor axonal neuropathy. No abnormality was observed on general laboratory tests.

Patient 2. A 25-year-old male patient presented with foot deformity, recurrent falls, and bilateral foot drop, which started at the age of 10 years. The patient reported a family history of consanguinity, with his parents being third cousins and his great-grandparents being first cousins. A neurological examination revealed high-arched feet, hammer toes, scoliosis, distally-predominant weakness in the upper limbs, fine postural tremor of the hands, and decreased proprioceptive sensation in the lower limbs. The reflexes were globally absent, except for those of the patella, which were normal. Electroneuromyography demonstrated symmetrically-decreased motor conduction amplitude, primarily suggestive of axonal compromise and signs of active denervation. A nerve biopsy exhibited no specific abnormalities, with only a mild increase in the endoneurial connective tissue observed. The levels of creatine kinase (CK) were mildly elevated, but no other serum laboratory tests were found to be abnormal.

Patient 3. A 72-year-old woman presented with slowly-progressive weakness in her lower limbs that had started at the age of 10 years. At 45 years, she required assisted ambulation, with the upper limbs also affected. Inability to walk and bulbar symptoms were present at 59 years old. There was moderate weakness in the hands, with intrinsic muscle atrophy; moderate distal and mild proximal weakness was found in the lower limbs. The electrophysiological examination revealed motor axonal neuropathy. The muscle biopsy demonstrated slight variation in the caliber of the muscle fibers, with predominance of type I, and mitochondrial abnormalities. The patient's condition deteriorated, and she passed away at the age of 72 years due to infectious complications.

Patient 4. A 67-year-old woman, the sister of patient 3. She reported a lifelong progressive weakness since childhood, initially on her lower limbs, but affecting her upper limbs throughout the decades since the onset of the symptom. At the age of 65 years, she was in a wheelchair after a traumatic brain injury, with contusion and intraparenchymal hematoma due to falling from her height. There was tetraparesis predominantly affecting the lower limbs, distal muscle atrophy in the four limbs, pes cavus, spastic hypertonia, and hyperreflexia in the upper limbs, accompanied by tendon retraction ( Figure 1F,G ). The electrophysiological study revealed a motor axonal neuropathy similar to that of patient 3. The muscle biopsy was suggestive of mitochondrial myopathy and chronic reinnervation, with variation in the diameter of the muscle fibers, slight disarrangement of the intermyofibrillar cytoarchitecture, with predominance of type I, and some areas of fiber type grouping ( Figure 1J–M ). A brain MRI scan revealed extensive microangiopathy (in the periventricular and deep white matter, classified as grade 3 on the Fazekas scale) ( Figure 1C,D ). There was no hypertension or diabetes, and the patient denied smoking.

Patient 5 is a 14-year-old female adolescent, granddaughter of patient 4. She presented a history of frequent falls and pain in the lower limb after exertion, starting at the age of 10 years. The mother confirmed mild intellectual disability and inattention early in childhood. The clinical examination revealed weakness in both proximal and distal muscles of the upper limbs and high-arched feet ( Figure 1H ). The serum laboratory tests were regular, except for slightly elevated CK (214 U/L). Electroneuromyography revealed motor axonal neuropathy with active denervation.

Patients 3, 4, and 5 had a family history of consanguinity (parents who were second cousins) and were from a small town in Brazil where consanguineous marriages were not rare.

Since patients 3 and 4 presented elevated serum CK levels and weakness, they underwent muscle biopsy at the time for differential diagnosis of mitochondrial disease, at a time when genetic testing was not yet available.

Except for patient 2, the remaining cases received CoQ10, at daily doses ranging from 200 to 400 mg for 1 or 2 years. No clear improvement or worsening was noted in any patient. The five patients underwent a genetic panel for neuromuscular disorders, and the p.Met1? variant of the *COQ7* gene (chr16:19,067,667 G > T; c.3G > T - ENST00000321998) was identified in homozygosity in all reported cases.

### Narrative review

A broad spectrum of phenotypic manifestations is associated with *COQ7* deficiency, ranging from pure neurological forms to an MF. The most well-known form of MF related to *COQ7* used to be a progressive encephalo-neuro-nephro-cardiopathy, frequently presenting with tubulopathy, lactic acidosis, hypertrophic cardiomyopathy, growth retardation, and delayed motor development. 6 11 17 28 29 So far, this form has been reported to be associated with loss-of-function missense or frameshift variants. Both pure and complex forms of hereditary spastic paraplegia (HSP) have also been described in association with missense pathogenic *COQ7* variants (3 cases with p.Leu111Pro, 3 cases with p.Arg54Gln, and 1 case with p.Pro108Thr). 13 14 18 19 29 In the last few years, cases associated with the axonal phenotype Charcot-MarieTooth (CMT) disease were published: 11 12 15 1 case with c.319C > T; Arg107Trp, one case with c.467T > G (p.Leu156Arg) and c.599_600delAGins TAATGCATC (p.Lys200IlefsTer56), and 1 case with c.446A > G;(p.Tyr149Cys) and c.3G > T (p.Met1?).

A Brazilian case of the *COQ7* dHMN phenotype with the homozygous variant p.Met1? (c.1A > G) was presented as a poster at a national congress in 2021. 23 In 2023, Smith et al. 18 reported a consanguineous Syrian family with 3 affected siblings, all with the dHMN phenotype associated with the same biallelic variant. Jacquier et al. 21 reported a Portuguese family with 3 dHMN cases associated with the biallelic variant p.Met1? (c.3G > T). In both studies, the variant's pathogenicity was demonstrated in in-vitro studies, which showed a severe decrease in COQ7 protein levels in the probands' fibroblasts and a decrease in CoQ10 production. In 2023, similar Brazilian cases were described related to p.Met1?: 2 unrelated women with infant-onset dHMN (presented as a conference poster), 25 a case of youth-onset dHMN, 10 11 1 case of the juvenile amyotrophic lateral sclerosis (ALS) form, 16 and Rebelo et al. 15 reported 6 cases in 5 Brazilian families with dHMN. Other dHMN variants were reported: c.161G > A;(p.Arg54Gln), 10 15 c.253-2A > T/c.467T > A, 22 c.160C > T/c.467T > G, 22 c.197T > A (Ile66Asn)/c.446A > G (Tyr149Cys), 24 c.197T > A (Ile66Asn)/c.319C > T (p.Arg107Trp), 15 and c.197T > A (p.Ile66Asn) and c.446A > G (p.Tyr149Cy). 15

**Figure 2:**
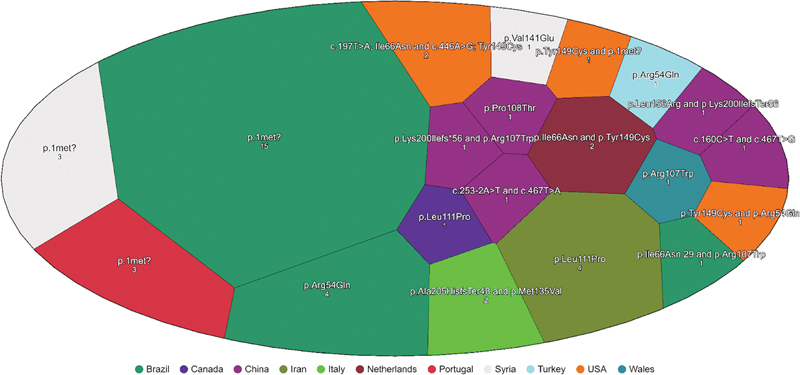
Voronoi map of *COQ7* cases by country and variant.

The different variants with phenotype and origin correlation are presented in Table 1 and illustrated in a Voronoi map in Figure 2 .

**Table 1:** COQ7 cases categorized by phenotype, variant, inheritance pattern and country

n°	Phenotype	Variant	Pattern	Region	Reference
1	dHMN	c.3G > T (p.1met?)	B	Brazil	Present study
2	dHMN	c.3G > T (p.1met?)	B	Brazil	Present study
3	dHMN	c.3G > T (p.1met?)	B	Brazil	Present study
4	dHMN	c.3G > T (p.1met?)	B	Brazil	Present study
5	dHMN	c.3G > T (p.1met?)	B	Brazil	Present study
6	dHMN	c.3G > T (p.1met?)	B	Brazil	Rebelo et al. 15
7	dHMN	c.3G > T (p.1met?)	B	Brazil	Rebelo et al. 15
8	dHMN	c.3G > T (p.1met?)	B	Brazil	Rebelo et al. 15
9	dHMN	c.3G > T (p.1met?)	B	Brazil	Rebelo et al. 15
10	dHMN	c.3G > T (p.1met?)	B	Brazil	Rebelo et al. 15
11	dHMN	c.3G > T (p.1met?)	B	Brazil	Rebelo et al. 15
12	dHMN	c.3G > T (p.1met?)	B	Brazil	Souza et al. 20
13	dHMN	c.3G > T (p.1met?)	B	Brazil	Wongkittichote et al. 11
14	SPG	c.161G > A	B	Brazil	Santos and Légora 23
15	SPG	c.161G > A;(p.Arg54Gln)	B	Brazil	Wongkittichote et al.
16	dHMN	c.161G > A;(p.Arg54Gln)	B	Brazil	Wongkittichote et al. 11
17	dHMN	c.197T > A; Ile66Asn 29 and c.319C > T; p.Arg107Trp	CH	Brazil	Rebelo et al. 15
18	dHMN	c.161G > A; p.Arg54Gln	B	Brazil	Rebelo et al. 15
19	dHMN	p,1met?	B	Brazil	Alvarenga et al. 27
20	dHMN	p,1met?	B	Brazil	Alvarenga et al. 27
21	SPG	c.332T > C (p.Leu111Pro)	B	Canada	Wang et al.
22	SPG	c.322C > A (p.Pro108Thr)	B	China	Qiu et al. 14
23	CMT2	p.Leu156Arg and p.Lys200IlefsTer56	CH	China	Zhang et al. 12
24	dHMN	c.253-2A > T and c.467T > A	CH	China	Liu et al. 22
25	dHMN	c.160C > T and c.467T > G	CH	China	Liu et al. 22
26	MF	p.(Lys200Ilefs*56) and p.(Arg107Trp)	CH	China	Kwong et al. 17
27	MF	c.332 T > C; p.(Leu111Pro)	B	Iran	Wang et al. 5
28	SPG	c.332T > C;p.Leu111Pro	B	Iran	Hashemi et al. 19
29	SPG	c.332T > C;p.Leu111Pro	B	Iran	Hashemi et al. 19
30	SPG	c.332T > C;p.Leu111Pro	B	Iran	Sadr et al. 13
31	MF	p.Ala205HisfsTer48 and p.Met135Val	CH	Italy	Pettenuzzo et al. 28
32	MF	p.Ala205HisfsTer48 and p.Met135Val	CH	Italy	Pettenuzzo et al. 28
33	dHMN	p.Ile66Asn and p.Tyr149Cys	CH	Netherlands	Theunissen et al. 24
34	dHMN	p.Ile66Asn and p.Tyr149Cys	CH	Netherlands	Theunissen et al. 24
35	dHMN	c.3G > T (p.1met?)	B	Portugal	Jacquier et al. 21
36	dHMN	c.3G > T (p.1met?)	B	Portugal	Jacquier et al. 21
37	dHMN	c.3G > T (p.1met?)	B	Portugal	Jacquier et al. 21
39	MF	p.Val141Glu	B	Syria	Freyer et al. 6
40	dHMN	c.1A > G (p.1met?)	B	Syria	Smith et al. 18
41	dHMN	c.1A > G (p.1met?)	B	Syria	Smith et al. 18
42	dHMN	c.1A > G (p.1met?)	B	Syria	Smith et al. 18
43	SPG	p.Arg54Gln	B	Turkey	Wang et al. 29
44	dHMN	p.Ile66Asn and p.Tyr149Cys	CH	USA	Rebelo et al. 15
45	dHMN	p.Ile66Asn and p.Tyr149Cys	CH	USA	Rebelo et al. 15
46	MF	p.Tyr149Cys and p.Arg54Gln	CH	USA	Wongkittichote et al. 11
47	CMT2	p.Tyr149Cys and p.1met?	CH	USA	Wongkittichote et al. 11
48	CMT2	p.Arg107Trp	B	Wales	Rebelo et al. 15

Abbreviations: B, biallelic; CH, compound heterozygous; CMT2, Charcot-Marrie-Tooth disease type 2; dHMN, distal hereditary motor neuropathy; MF, multisystemic form; SPG, spastic paraplegia; USA, United States of America.

### Statistical analysis

**Figure 3:**
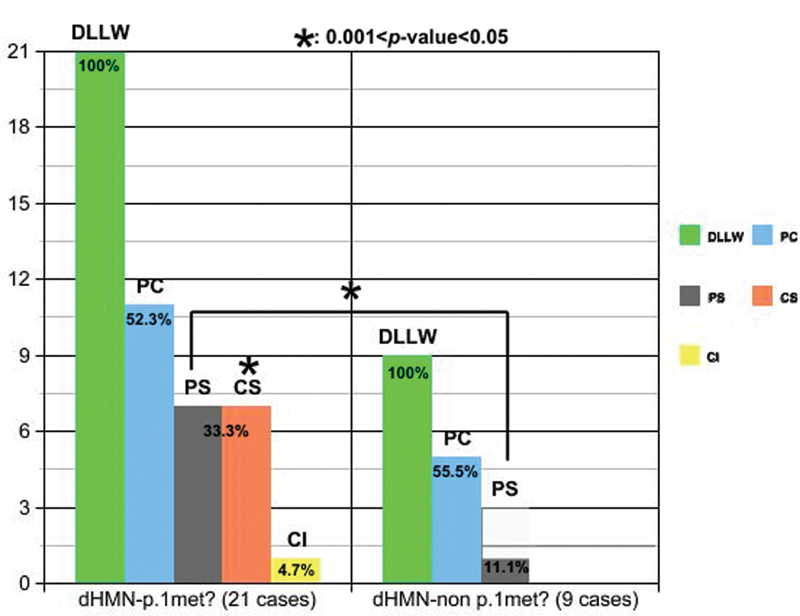
( **A** ) Percentage of the phenotype in cases of Coenzyme Q10 deficiency-8 CoQ10D8 associated with the biallelic p.Met1 variant?. ( **B** ) Percentage of the phenotype in cases of CoQ10D8 associated with other variants reported in the literature.

In our search, we found 20 studies from 10 countries involving 47 patients with *COQ7* -related disorders. In total, 30 out of the 47 cases were related to dHMN (63.8%), 8, to SPG (17%), 6, to MF (12.7%), and 3, to CMT (6.3%). The p.Met1? variant was present in 21 *COQ7* cases (44.6%) ( Table 2 ). All patients presented dHMN: 9 (42.8% of the p.Met1? cases) with the pure dHMN phenotype, and 12 (57.2% of the p.Met1? cases) with dHMN plus. The dHMN plus cases were patients with the main clinical feature of motor axonal neuropathy but associated with other symptoms: 4 (19% of the p.Met1? cases) had dHMN associated with CA, 3 (14.2% of the p.Met1? cases) had dHMN and pyramidal signs (PS), another 3 (14%) had dHMN associated with CA and PS, 1 case (4.7% of the p.Met1? cases) was associated with cognitive impairment (the one reported in the present study), and 1 had juvenile ALS ( Figure 3A ). The origin of the p.Met1 cases were: 71.4% from Brazil, 14.2% from Portugal, and 14.2% from Syria. With the other variants, a high phenotypic heterogeneity was observed: 9 (34.6%) cases of dMHN (8 with pure and 1 with PS), 8 (30.7%) cases of SPG, 6 (23%) cases of MF, and 3 (11.5%) cases of CMT ( Figure 3B ). Regarding the Brazilian dHMN cohorts, 2 studies 25 26 were included, amounting to 170 cases, with no *COQ7* disease reported among them.

**Table 2:** dHMN related to p.1met? *COQ7* cases in literature

Family	FH	Sex	age	Region	Age of Onset	PULW	DULW	PLLW	DLLW	PS	CI	CS	Reference
I	+	M	?	Syria	10	−	+	−	+	−	−	−	Smith et al. 18
I	+	F	?	Syria	10	−	+	−	+	−	−	−	Smith et al. 18
I	+	M	?	Syria	10	−	+	−	+	−	−	−	Smith et al. 18
II	+	F	36	Portugal	12	−	+	−	+	+	−	−	Jacquier et al. 21
II	+	F	25	Portugal	9	−	+	−	+	+	−		Jacquier et al. 21
II	+	M	42	Portugal	10	−	−	+	+	−	−	−	Jacquier et al. 21
III	+	F	43	Brazil	5	−	+	−	+	+	−	+	Rebelo et al. 15
III	+	F	39	Brazil	10	−	+	−	+	+	−	+	Rebelo et al. 15
IV	−	F	52	Brazil	1	−	−	−	+	−	−	+	Rebelo et al. 15
V	−	F	56	Brazil	Childhood	−	−	−	+	−	−	+	Rebelo et al. 15
VI	−	M	50	Brazil	4	−	−	−	+	−	−	+	Rebelo et al. 15
VII	−	F	52	Brazil	?	−	−	−	+	−	−	−	Rebelo et al. 15
VIII	+	M	38	Brazil	11	−	+	+	+	+	−	−	Souza et al.
IX	−	F	33	Brazil	5	−	+	−	+	+	−	+	Wongkittichote et al.
X	−	F	34	Brazil	Puberty	−	−	−	+	−	−	−	Alvarenga et al. 27
XI	+	F	16	Brazil	Puberty	−	−	−	+	−	−	−	Alvarenga et al. 27
XII	−	M	40	Brazil	Childhood	+	+	−	+	+	−	−	Present study
XIII	−	M	25	Brazil	10	−	+	−	+	−	−	−	Present study
XIV	+	F	72	Brazil	Childhood	+	+	+	+	−	−	−	Present study
XIV	+	F	67	Brazil	Childhood	+	+	+	+	−	−	−	Present study
XIV	+	F	14	Brazil	10	+	−	+	+	−	+	+	Present study

Abbreviations: CI, cognitive impairment; CS, cerebellar syndrome; DLLW, distal lower-limb weakness; DULW, distal upper-limb weakness; F, female; FH, family history; M, male; PLLW, proximal lower-limb weakness; PS, pyramidal sign; PULW, proximal upper-limb weakness.

**Figure 4:**
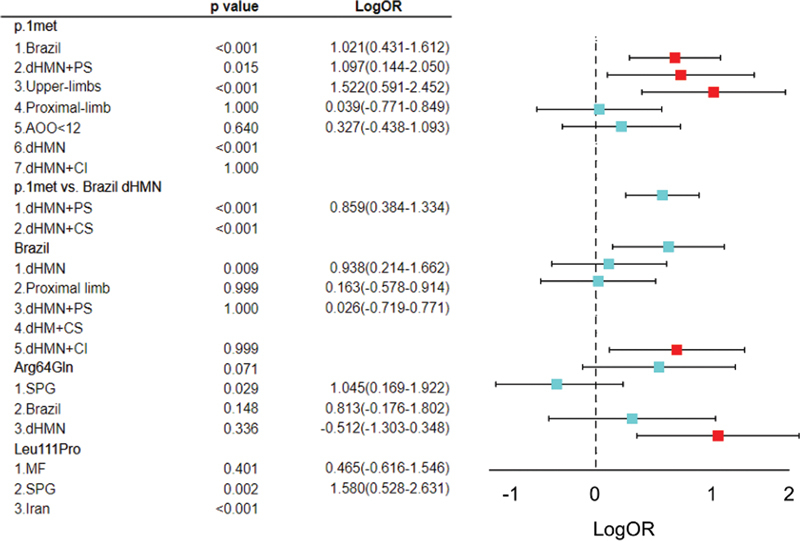
Values of *p* and LogOR (odds ratio) (Log10[Lower Odds Ratio] - Log10[Upper Odds Ratio]) of the categorical variables on the left side and forest plot on the right side.

On the comparison of the phenotypes among the presence of variants, the p.Met1? was significantly more frequent in patients with dMHN, considering all cases of dHMN (pure and plus; 21 out 30; *p* -value < 0.001) ( Figure 3 ). Brazilian origin was also significantly related to the p.Met1? variant compared to other regions (15 out 21; *p* -value < 0.001; OR = 10.5; 95%CI = 2.696–40.880). Brazilians have a statistically significant relationship with the dHMN phenotype (13 out of 28; *p* -value = 0.009; OR = 8.6; 95%CI = 1.63–45.86). All CA patients were from Brazil; this relationship was also statistically significant ( *p* -value = 0.0063; Figure 4 ).

Compared to the 170 cases of several types of dHMN from Brazil, 25 26 PS ( *p* -value < 0.001; OR = 7.2273; 95%CI = 2.4205–21.5797) and CA ( *p* value < 0.001) associated with dHMN were statistically significant for p.Met1? *COQ7* ( Figure 3 ). The SPG- *COQ7* variant is related to p.Leu111Pro, with statistical significance ( *p* -value = 0.00181; OR = 34; 95%CI = 3.3741–427.9693). This variant had a strong association with Iran ( *p* -value < 0.001). The p.Arg54Gln variant was statistically associated with SPG ( *p* -value = 0.029; OR = 11.1; 95%CI = 1.475–83.5322) ( Figure 4 ).

We also performed multivariate statistical analyses based on penalized logistic regression models, for each clinical outcome of interest and fitted separate logistic regression models using genotype and country of origin as predictors. The variants were binarized according to their presence or absence in each individual (p.Met1?, p.Arg54?, p.Leu111Pro), and geographic origin was coded as *Brazil* versus *non-Brazil* . Given the limited sample size and the presence of sparse cells in the contingency tables, a penalty term (L2-regularization) was applied to stabilize the coefficient estimation and reduce small-sample bias. The models were fitted using maximum likelihood estimation with ridge penalization and a fixed regularization parameter (C = 1.0), and effect sizes were derived as adjusted ORs. To obtain empirical 95%CIs, we generated bootstrap resampling distributions (500 iterations per model), computing percentile-based confidence limits for each predictor. Because of the sparse patterns in several outcomes, the results were considered exploratory and hypothesis-generating rather than confirmatory. The p.Met1? variant and Brazilian origin remained associated with selected phenotypic features after the multivariable adjustment, although the 95%CIs remained wide. These models were used strictly to test whether the observed univariate associations persisted after mutual adjustment among predictors. Complete data on the statistical analysis can be found in Tables 3 and 4 .

**Table 3:** Variable correlations - phenotype, variant of COQ7gene and patient's origin

Variant	OR	LOR	UOR	*p* -value	LogOR	LogLOR	LogUOR
p.1met? and dHMN				< 0.001			
p.1met? and Brazil	10.5	2.6969	40.8804	< 0.001	1.021189299	0.4308648433	1.611515137
p.1met? and PS	8.8	0.929	112.2612	1.0	0.9444826722	−0.03198428601	2.05022968
p.1met? and CS				0.071			
p.met? and PS compared to other dHMN from Brazil	7.2273	2.4205	21.5797	< 0.001	0.8589760823	0.383905087	1.334045403
p.1met? and cs compared to other dHMN from Brazil				< 0.001			
p.1met? and CI				1.000			
p.1met? and ULW	33.25	3.9002	283.4647	0.001	1.52179165	0.591086878	2.452498984
p.1met? and PLW	1.0938	0.169	7.0608	1.000	0.03893791904	−0.7721132954	0.8488539101
p.1met? and AO < 12 years-old	2.125	0.365	12.3851	0.640	0.3273589344	−0.4377071355	1.092899518
Brazil and dHMN	8.6667	1.638	45.8686	0.009	0.9378537636	0.2143138974	1.661515485
Brazil and CS				0.006			
Brazil and CI				0.999			
Brazil and PLW	1.4545	0.264	8.1997	0.999	0.1627137256	−0.5783960731	0.9137979633
Brazil and PS	1.0606	0.191	5.9032	1.000	0.02555162278	−0.7189666328	0.7710874973
p.Arg and SPG	11.1	1.475	83.5322	0.029	1.045322979	0.1687920203	1.92185392
p.Arg and Brazil	6.5	0.666	63.4294	0.148	0.8129133566	−0.1765257708	1.802290603
p.Arg and dHMN	0.3077	0.0498	2.2304	0.336	−0.5118725038	−1.302770657	0.3483827564
p.Leu111Pro and SPG	38	3.3741	427.9693	0.00181	1.579783597	0.5281579499	2.631412616
p.Leu111Pro and Iran				< 0.001			
p.Leu111Pro and MF	2.916	0.2422	35.123	0.401	0.4647875196	−0.6158258612	1.545591604

Abbreviations: AO, age at onset; CI, cognitive impairment; CS, cerebellar sign; dHMN, distal hereditary motor neuropathy; LogLOR, logarithmic value of the LOR; LogOR, logarithmic value of the OR; LogUOR, logarithmic value of the UOR; LOR, lower value of the OR; MF, multisystemic form; OR, odds ratio; PLW, proximal limb weakness; PS, pyramidal sign; SPG, spastic paraplegia; ULW, upper limb weakness; UOR, upper value of the OR.

**Table 4:** Multivariate penalized logistic regression results (L2), adjusted for genotype and geographic origin

Outcome	Predictor	OR	95%CI	n
PS	p.Met1?	3.52	0.88–14.01	13
PS	p.Arg54?	1.11	0.32–4.75	13
PS	p.Leu111Pro	0.86	0.18–4.03	13
PS	Brazil	2.09	0.57–8.34	13
CS	p.Met1?	4.28	0.96–23.65	13
CS	p.Arg54?	0.91	0.24–4.07	13
CS	p.Leu111Pro	0.73	0.15–3.60	13
CS	Brazil	3.64	1.02–14.55	13
CI	p.Met1?	5.21	1.03–21.73	13
CI	p.Arg54?	1.06	0.30–4.40	13
CI	p.Leu111Pro	1.54	0.30–8.22	13
CI	Brazil	2.01	0.41–8.88	13
ULW	p.Met1?	1.94	0.46–6.91	13
ULW	p.Arg54?	1.12	0.23–5.27	13
ULW	p.Leu111Pro	0.77	0.20–3.65	13
ULW	Brazil	2.84	0.54–10.71	13
PLW	p.Met1?	3.31	0.88–15.27	13
PLW	p.Arg54?	1.32	0.29–6.74	13
PLW	p.Leu111Pro	0.59	0.12–2.97	13
PLW	Brazil	4.18	1.03–22.66	13
AO < 12 years	p.Met1?	6.18	1.25–31.73	13
AO < 12 years	p.Arg54?	0.97	0.23–3.69	13
AO < 12 years	p.Leu111Pro	0.81	0.16–3.41	13
AO < 12 years	Brazil	3.95	0.98–20.61	13

Abbreviations: AO, age at onset; CI, cognitive impairment; CS, cerebellar syndrome; OR, odds ratio; PLW, proximal lower-limb weakness; PS, pes cavus/structural foot change; ULW, upper-limb weakness.

## DISCUSSION

Although *COQ7* deficiency can lead to different phenotypes, we herein reported 5 patients from 3 different kinships, all with dHMN. Until 2024, this presentation was not featured among the *COQ7* spectrum, and it could be considered an uncommon manifestation of the disease. 19 30 Among dHMNs, neither was *COQ7* considered a possible etiology until recently, not even as a rare etiology for dHMNs. 31 However, a new scenario with distal motor axonal neuropathy has been reported in several individuals with *COQ7* -related CoQ10 deficiency. 12 13 15 18 19 20 21 22 23 The 5 additional cases herein reported strengthen the notion that this presentation is a crucial phenotype linked to the gene. This phenotype has now become the most reported phenotype related to *COQ7* in the current literature, with or without concomitant involvement of the first motor neuron.

The p.Met1? (c.3G > T and c.1A > G) variant was found to be statistically linked to the dHMN phenotype ( *p* < 0.001).All patients homozygous for this variant had dHMN: 9 (42.8% of the p.Met1? cases) with the pure dHMN phenotype and 12 (57.2% of the p.Met1? cases) with dHMN plus. This suggests that the clinical manifestations of *COQ7* -related CoQ10 deficiency may be correlated to specific variants, possibly varying according to the locus and type of the variant. The p.Met1? variant disrupts the translation initiation codon, leading to a loss of protein production. Thus, protein synthesis is prevented from beginning at the predicted point. A significantly-reduced translation of *COQ7* isoform 1 was already demonstrated with this variant. 21 Other isoforms of the protein can probably provide mitochondria with *COQ7* , which would partially compensate for the loss of isoform 1 with some grade of phenotype rescue. Another reported variant in the same codon (c.1A > G; p.Met1?) was also associated with pure dHMN, supporting this view. Thus, the hypothesis raised by Jacquier et al. 21 in 2023 is reinforced here: restricted dHMN would be a milder presentation related to p.Met1?, due to its lower impact compared to variants located in the catalytic site. 21

Patient 1 presented dHMN plus with cerebellar and pyramidal syndrome with cerebellar atrophy on MRI ( Figure 1C,D ). Patient 2 had pure dHMN. Patients 3 and 4, siblings, both had dHMN plus (PS), and patient 5 had dHMN plus with cognitive impairment. All patients presented onset of symptoms in the first decade of life. These findings are consistent with those of other reports of dHMN related to *COQ7* in the literature. The phenotype with dHMN associated with CA was observed in 7 cases in the literature, all harboring the p.Met1? variant. However, CA was only reported in patients of Brazilian origin, which was also significantly linked to dHMN with CA ( *p* < 0.001), Moreover, PS in Brazilian patients with dHMN was associated with *COQ7* . Therefore, among Brazilian patients, dHMN with CA and/or PS suggests a higher chance of *COQ7* deficiency. These findings raise the hypothesis that the environment could also play a significant role in phenotype expression.

The neuroimaging features related to *COQ7* deficiency were only reported in MF: hyperintensity in the periventricular white matter on T2-weighted and fluid-attenuated inversion recovery (FLAIR) sequences, multiple cystic changes involving bilateral corona radiata, the basal ganglia, and thalami, brainstem hypoplasia, mega cisterna magna, severe thinning of the corpus callosum, enlargement of the ventricular system, and progressive cortical atrophy. 28 32 In the present study, Patient 4, with dHMN and PS, had a prominent periventricular and deep white matter T2 hyperintensity, besides her age and absence of other microangiopathy risk factors. This image finding would be worthy of search on other cases of *COQ7* deficiency, to verify if could be another milder manifestation of the disease, or even of the specific p.Met1? variant.

Except for patient 3, our cases were put on CoQ10 reposition. Neither improvement nor deterioration was found in this short observation period. Furthermore, all treated cases started on CoQ10 late in life and at low dosages (100–500 mg a day, due to the high cost), which hinders the observation of benefits. Oral CoQ10 treatment has been partially successful in some cases of primary CoQ10 deficiency. 25 33 Nevertheless, no apparent improvement in *COQ7* patients has been indisputably observed. 11 15 This treatment presents deficient absorption and bioavailability due to high molecular weight and low aqueous solubility, which limits its therapeutic potential. 34 Consensus regarding the dose of the CoQ10 replacement is lacking, and the recommended dosage ranges from 5 to 50 mg/kg/day, with 1,200mg a day as the maximum safest dose. 33 Higher doses of CoQ10, with early beginning of treatment, could lead to better responses once in-vitro studies suggest improvement with the replacement. 15 21 Furthermore, patients with peripheral manifestations may exhibit a better therapeutic response than those with central or multisystemic involvement. 35

In conclusion, the present study supports the notion that *COQ7* is an important cause of dHMN. The cases herein reported and data in the literature suggest that specific *COQ7* variants may be associated with specific phenotypes: p.Met1?, with dHMN, and p.Arg54Gln and p.Leu111Pro, with SPG. Additionally, the p.Met1? variant seems to be linked to CA as well, especially in cases of Brazilian origin. Among Brazilian patients, dHMN with CA and/or PS suggests a higher chance of *COQ7* deficiency. In Brazilian patients, CA raises the hypothesis of environmental influence in disease expression. Finally, white matter hyperintensity on MRI can also manifest in mild *COQ7* cases. Even after multivariable adjustment for genotype distribution and geographic origin, the p.Met1? variant remained associated with earlier age at onset, cognitive impairment, and proximal lower-limb weakness, reinforcing its relevance as a phenotypic driver within *COQ7* -related disorders. Likewise, Brazilian origin continued to demonstrate an independent association with cerebellar involvement, suggesting the influence of contextual or modifying factors that extend beyond genotype alone. Although these findings were derived from exploratory penalized models and the 95%CIs remained wide due to the small sample size, they provide additional support for genotype–phenotype clustering and highlight the need for larger, systematically-characterized cohorts to validate these observations.
